# Bisphenol A at Low Nanomolar Doses Confers Chemoresistance in Estrogen Receptor-α–Positive and –Negative Breast Cancer Cells

**DOI:** 10.1289/ehp.11788

**Published:** 2008-10-08

**Authors:** Elizabeth W. LaPensee, Traci R. Tuttle, Sejal R. Fox, Nira Ben-Jonathan

**Affiliations:** Department of Cancer and Cell Biology, University of Cincinnati, Cincinnati, Ohio, USA

**Keywords:** bisphenol A, breast cancer cells, chemotherapeutic agents, cytotoxicity, estrogen receptors

## Abstract

**Background:**

Resistance to chemotherapy is a major problem facing breast cancer patients, and identifying potential contributors to chemoresistance is a critical area of research. Bisphenol A (BPA) has long been suspected to promote carcinogenesis, but the high doses of BPA used in many studies generated conflicting results. In addition, the mechanism by which BPA exerts its biological actions is unclear. Although estrogen has been shown to antagonize anticancer drugs, the role of BPA in chemoresistance has not been examined.

**Objective:**

The objective of our study was to determine whether BPA at low nanomolar concentrations opposes the action of doxorubicin, cisplatin, and vinblastine in the estrogen receptor-α (ERα)-positive T47D and the ERα-negative MDA-MB-468 breast cancer cells.

**Methods:**

We determined the responsiveness of cells to anticancer drugs and BPA using the 3-(4,5-dimethylthiazol-2-yl)2,5-diphenyl tetrazolium bromide (MTT) cytotoxicity assay. Specific ERα and ERβ inhibitors and real-time polymerase chain reaction were used to identify potential receptor(s) that mediate the actions of BPA. Expression of antiapoptotic proteins was assessed by Western blotting.

**Results:**

BPA antagonizes the cytotoxicity of multiple chemotherapeutic agents in both ERα-positive and -negative breast cancer cells independent of the classical ERs. Both cell types express alternative ERs, including G-protein–coupled receptor 30 (GPR30) and members of the estrogen-related receptor family. Increased expression of antiapoptotic proteins is a potential mechanism by which BPA exerts its anticytotoxic effects.

**Conclusions:**

BPA at environmentally relevant doses reduces the efficacy of chemotherapeutic agents. These data provide considerable support to the accumulating evidence that BPA is hazardous to human health.

Bisphenol A (BPA) is a monomer of polycarbonate plastics that is used in numerous consumer products, including food and water containers, baby bottles, linings of metal food and beverage cans, medical tubing, epoxy resins, and dental fillings (Welshons et al. 2006). Small amounts of BPA can be liberated from incompletely polymerized polycarbonates or via partial hydrolysis, especially upon heating (Le et al. 2008). Decades of continuous release of free BPA into food, beverages, and the environment have resulted in a widespread human exposure to this chemical. Many studies in the United States, Europe, and Japan have documented BPA levels ranging from 0.2 to 10 ng/mL (~ 0.5–40 nM) in adult and fetal human serum (Welshons et al. 2006) as well as in breast milk (Kuruto-Niwa et al. 2007). Being lipophilic, BPA can also accumulate in fat, and detectable levels of BPA have been found in half of breast adipose tissue samples examined (Fernandez et al. 2007).

Given the structural similarity of BPA to the potent estrogenic compound diethyl-stilbestrol, BPA’s ability to promote carcinogenesis has long been suspected (Keri et al. 2007). Studies with rodents have revealed that early-life exposure to BPA causes increased susceptibility to mammary and prostate tumorigenesis (Prins et al. 2007; Soto et al. 2008), but there is less evidence for carcinogenic activity of BPA when administered to adult animals. Studies with human breast cancer cells have yielded inconsistent data with respect to the mitogenic, apoptotic, and transcriptional properties of BPA (Dairkee et al. 2008; Diel et al. 2002; Singleton et al. 2006; Soto et al. 1995). This inconsistency is attributed to the wide variations in BPA doses used, some of which are at micromolar levels. BPA often exhibits a U-shaped or an inverted U-shaped dose–response curve. Consequently, extrapolation from an action, or lack of action, of BPA at high doses to its presumed bioactivity at low doses is unwarranted. Thus, to support the argument that BPA poses risks to human health, it is necessary to establish its effectiveness at environmentally relevant concentrations (the low nanomolar range).

The mechanism by which BPA exerts it biological actions is enigmatic. The binding affinity of BPA to estrogen receptor-α (ERα) or ERβ is 10,000- and 1,000-fold lower than that of estradiol (E_2_), respectively (Kuiper et al. 1998). This suggests that BPA should mimic or compete with endogenous estrogens only at the micromolar range. Yet, BPA at nanomolar doses often displays activities that are similar to those of E_2_ (Watson et al. 2005; Welshons et al. 2006). To reconcile this dilemma, several speculations have been proposed. One view is that BPA binds differently within the ligand-binding domain of ERα or ERβ and recruits a dissimilar set of co-regulators (Safe et al. 2002). Other investigators maintain that BPA elicits its responses via non classical ERs, including membrane-anchored ERs (Watson et al. 2005), G-protein–coupled receptor 30 (GPR30; Thomas and Dong 2006), and members of the estrogen-related receptors (ERRs) such as ERRγ, which has a high binding affinity to BPA (Okada et al. 2008).

Although most studies to date have examined whether BPA stimulates breast cancer cell proliferation, its potential effects on chemotherapeutic efficacy have received little attention. Chemotherapy, alone or in combination with hormonal or targeted therapy, remains the mainstay treatment in metastatic breast disease. A wide variety of anticancer drugs are available, including doxorubicin, cisplatin, and vinblastine. Most regimens combine agents that act by different mechanisms to improve efficacy. Although treatment of breast cancer patients with these anticancer drugs has shown good success, tumor resistance remains a major obstacle. Some tumors are intrinsically resistant to certain drugs, whereas others can acquire resistance after treatment. Although the effects of environmental pollutants on drug transporters as well as on metabolic and detoxifying enzymes have been explored to some extent (Brockmoller et al. 2000; Chen et al. 1998; Han and Zhang 2004), there is no information on whether endocrine disruptors can modulate the responsiveness of breast cancer cells to anti-cancer drugs.

The objectives of this study were to *a* ) compare the effects of low doses of BPA on cisplatin, doxorubicin, and vinblastine cytotoxicity in the estrogen-responsive T47D breast cancer cells; *b* ) examine whether BPA exerts similar effects on the estrogen-insensitive MDA-MB-468 breast cancer cells; *c* ) compare expression of classical (ERα and ERβ) and nonclassical (GPR30, ERRα, ERRβ, and ERRγ) ERs in the two cell lines; *d* ) determine the effects of the ER antagonist ICI182,780 (ICI) and the ERβ-specific antagonist 4-[2- phenyl-5,7-bis(trifluoromethyl)pyrazolo[1,5-*a*] pyrimidin-3-yl]phenol (PHTPP) on the ability of BPA to antagonize the cytotoxic effects of doxorubicin; and *e*) examine whether the chemoresistant effects of BPA are mediated by altered expression of antiapoptotic/proapoptotic proteins of the Bcl-2 and survivin families.

## Materials and Methods

### Drugs and inhibitors

Doxorubicin (Sigma, St. Louis, MO), cisplatin (Sigma), and vinblastine (Biomol, Plymouth Meeting, PA) were dissolved in water at stock concentrations of 1 mg/mL (doxorubicin and cisplatin) or 0.1 mg/mL (vinblastine). ICI and PHTPP (both from Tocris Bioscience, Ellisville, MO) were dissolved in dimethyl sulfoxide (DMSO; 100 mM) and ethanol (50 mM), respectively. Drugs and inhibitors were diluted in culture medium immediately before treatment.

### Cell lines and culture conditions

We obtained T47D and MDA-MB-468 cells from the American Type Culture Collection (Manassas, VA). T47D cells were maintained in RPMI medium (Hyclone, Logan, UT) supplemented with 10% fetal bovine serum (FBS; Hyclone), 5 μg/mL bovine insulin, 10 mM HEPES, 1 mM sodium pyruvate, and 50 μg/mL normocin (InvivoGen, San Diego, CA). MDA-MB-468 cells were cultured in low-glucose Dulbecco’s modified Eagle’s medium (DMEM; Hyclone) supplemented with 10% FBS and 50 μg/mL normocin. For all experiments, T47D cells were plated in phenol red–free RPMI with 5% charcoal-stripped serum (CSS) and ITS+ supplement (1:200; BD Biosciences, Bedford, MA) and treated in RPMI with 3% CSS and ITS+. MDA-MB-468 cells were plated in phenol red–free DMEM supplemented with 3% CSS and treated in DMEM with 1% CSS.

### Cytotoxicity assay

Cells were plated at a density of 6,000 or 8,000 cells/well in 96-well plates in plating medium. The next day, cells were incubated with BPA for 24 hr in treatment medium. In the case of inhibitors, ICI and PHTPP were added to the cells 1 hr before BPA. After BPA treatment for 24 hr, the various drugs were added for an additional 1–4 days in the continuous presence of BPA. We determined cytotoxicity using 3-(4,5-dimethylthiazol-2-yl)2,5-diphenyl tetrazolium bromide (MTT). MTT was added at a final concentration of 0.5 mg/mL for 2 hr. After medium aspiration, the formazan dye was extracted with DMSO and absorbance was read at 570 nm using a plate reader (Bio-Tek, Winooski, VT).

### Western blotting

After treatment, we homogenized cells in buffer (10 nM Tris-HCl, 5 mM EDTA, 50 nM NaCl, 50 mM sodium fluoride, 30 mM sodium pyrophosphate, 1% Triton-X, 200 μM sodium orthovanadate, 1 mM phenyl methylsulfonyl fluoride, 1 μg/mL pepstatin, 2 μg/mL leupeptin, 5 μg/mL aprotinin). The protein concentration was determined using the Pierce (Rockford, IL) BCA (bicinchoninic acid) protein assay. Cell lysates (40 μg protein) were electrophoresed onto 12% or 15% sodium dodecyl sulfate polyacrylamide gel electrophoresis gels. After transfer to polyvinyl difluoride membranes, samples were blocked in 5% dry milk and incubated overnight with the following primary antibodies: Bcl-2, Bcl-xL, survivin (1:1,000 each; Cell Signaling, Danvers, MA), ERα (1:400; Santa Cruz Biotechnology, Santa Cruz, CA), ERβ (1:3,000; Upstate, Danvers, MA), or β-actin (1:10,000; Sigma). After incubation with horseradish peroxidase–conjugated secondary antibody (Amersham, Piscataway, NJ), products were developed on film using SuperSignal chemiluminescence reagents (Pierce).

### Real-time polymerase chain reaction (PCR)

Total RNA was isolated using Tri-Reagent (MRC, Cincinnati, OH) and cDNA was synthesized as previously described (Hugo et al. 2006). PCR was performed on 200 ng cDNA using intron-spanning primers for ERα, ERβ, GPR30, ERRα, ERRβ, and ERRγ; we used β2-microglobulin (β2M) as a reference gene. Primer sequences are listed in Table 1. We performed quantitative real-time PCR using Immolase heat-activated Taq DNA polymerase (Bioline, Taunton, MA). SYBR Green I (Invitrogen, Carlsbad, CA) was used for fluorometric product detection using a SmartCycler I cytometer (Cepheid, Sunnyvale, CA). Cycle parameters were 96°C for 15 min for polymerase activation, followed by 40 cycles of 95°C for 15 sec, 57°C for 15 sec, and 72°C for 30 sec, with an optical read stage at 83.5°C for 6 sec. We confirmed product purity by DNA melting curve analysis. After correction for β2M, fold changes in gene expression were calculated from the cycle threshold measurements as previously described (Pfaffl et al. 2002).

### Data analysis

Statistical differences were determined by one-way analysis of variance followed by Newman-Keuls post hoc analysis. *p*-Values < 0.05 were considered significant. All experiments were performed at least three times.

## Results

### BPA protects T47D cells from chemotherapeutic-induced cytotoxicity

**Figure 1:**
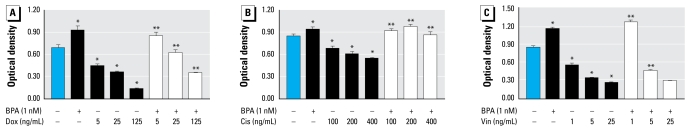
BPA protects T47D cells from several chemotherapeutic agents. Cells were treated with BPA for 24 hr, followed by increasing concentrations of doxorubicin (Dox; *A*), cisplatin (Cis; *B*), or vinblastine (Vin; *C*) for an additional 96 hr. Cytotoxicity was determined by the MTT assay. Values are mean ± SE of six replicates of a single experiment, repeated three times with similar results. *p < 0.05 compared with control. **p < 0.05 compared with the corresponding drug dose.

We first examined the sensitivity of the estrogen-responsive T47D cells to selected anticancer drugs, and determined whether BPA protects the cells from drug-induced cytotoxicity. As shown in Figure 1, doxorubicin induced a dose-dependent decrease in cell viability that was either completely or partially antagonized by a 24-hr pretreatment with a low dose of BPA (1 nM). The cells were less sensitive to cisplatin, with the highest tested dose (400 ng/mL) decreasing viability by approximately 40%. BPA prevented drug-induced cytotoxicity at all tested cisplatin doses. The cytotoxic effects of vinblastine on T47D cells resembled that of doxorubicin. Pretreatment with BPA was highly effective only against the lowest dose of vinblastine (1 ng/mL). In all cases, BPA alone increased cell viability.

### BPA antagonizes chemotherapeutic agents in MDA-MB-468 cells

**Figure 2:**
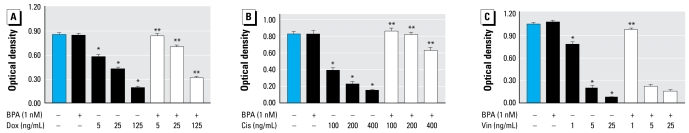
BPA antagonizes anticancer drugs in MDA-MB-468 cells. Cells were treated with BPA for 24 hr, followed by increasing concentrations of doxorubicin (Dox; *A*), cisplatin (Cis; *B*), or vinblastine (Vin; *C*) for an additional 96 hr. Cytotoxicity was determined by the MTT assay. Values are mean ± SE of six replicates of a single experiment, repeated three times with similar results. *p < 0.05 compared with control. **p < 0.05 compared with the corresponding drug dose.

We next examined whether BPA protected the estrogen-unresponsive MDA-MB-468 cells from the same anticancer drugs (Figure 2). Similar to T47D cells, doxorubicin treatment resulted in a dose-dependent decrease in MDA-MB-468 cell viability. BPA completely or partially protected the cells from all doses of doxorubicin. MDA-MB-468 cells were significantly more sensitive to cisplatin than were T47D cells, with the 400 ng/mL dose of cisplatin inhibiting cell viability by > 80%. All doses of cisplatin were antagonized by a pretreatment with BPA. BPA protected MDA-MB-468 cells only from the lowest dose of vinblastine. Unlike in T47D cells, BPA alone had no effect on cell viability.

### BPA, at low nanomolar concentrations, protects cells from doxorubicin-induced cytotoxicity

**Figure 3:**
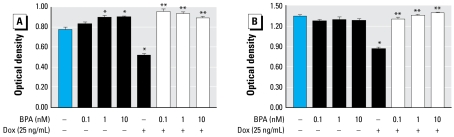
Low doses of BPA protect T47D (*A*) and MDA-MB-468 (*B*) cells from doxorubicin (Dox) treatment. Cells were treated with increasing doses of BPA for 24 hr, followed by doxorubicin for an additional 24 hr. Cytotoxicity was determined by the MTT assay. Values are mean ± SE of six replicates of a single experiment, repeated three times with similar results. *p < 0.05 compared with control. **p < 0.05 compared with doxorubicin.

The next experiment evaluated the ability of increasing, environmentally relevant doses of BPA to antagonize the cytotoxic effect of one dose of doxorubicin. Figure 3 shows that BPA alone (1 nM or 10 nM) significantly increased cell viability in T47D cells but not in MDA-MB-468 cells. In both cell types, doxorubicin treatment induced an approximately 35% decrease in cell viability. A 24-hr pretreatment with BPA at all doses examined completely protected the cells from doxorubicin-induced cytotoxicity.

### The protective effects of BPA are not mediated via classical ERs

**Figure 4:**
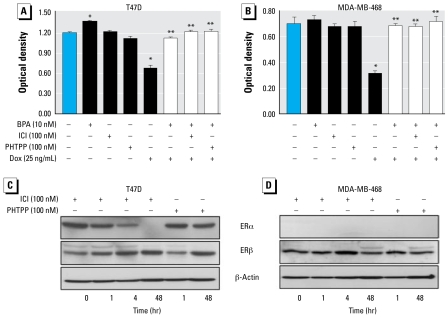
BPA mediates its protective effects independent of the classical ERs. T47D (*A*) and MDA-MB-468 (*B*) cells were treated with 100 nM ICI or PHTPP 1 hr before BPA (10 nM); after 24 hr pretreatment with BPA, cells were exposed to doxorubicin (Dox; 25 ng/mL) for an additional 24 hr. Cytotoxicity was determined by the MTT assay. Values are mean ± SE of six replicates of a single experiment, repeated three times with similar results. T47D (*C*) and MDA-MB-468 (*D*) cells were treated with 100 nM ICI or PHTPP for 1, 4, or 48 hr. Western blots were probed for ERα or ERβ; β-actin served as a loading control. Shown are representative blots, repeated at least three times. *p < 0.05 compared with control. **p < 0.05 compared with doxorubicin.

To determine if the protective effects of BPA involved ERα or ERβ, we used ICI, an antagonist of both receptors, as well as PHTPP, a specific ERβ antagonist. As shown in Figure 4A, neither ICI nor PHTPP had any effect by themselves on T47D or MDA-MB-468 cell viability. Furthermore, the ability of BPA to antagonize doxorubicin-induced cytotoxicity in either cell line was not altered in the presence of ICI or PHTPP. Using Western blotting, we next probed for both ERα and ERβ in T47D and MDA-MB-468 cells treated for 1, 4, or 48 hr with the above inhibitors. Figure 4B demonstrates that T47D cells, but not MDA-MB-468 cells, express ERα, whereas both cell types express ERβ. Treatment with ICI caused a time-dependent decrease in ERα expression in T47D cells, reducing it to an undetectable level by 48 hr. On the other hand, ERβ expression in MDA-MB-468 cells increased at 4 hr and decreased after 48 hr in response to ICI treatment. PHTPP had no effect on ERα, increased the expression of ERβ in T47D cells, and had no effect on ERβ in MDA-MB-468 cells.

### Relative receptor expression in T47D and MDA-MB-468 cells

**Figure 5:**
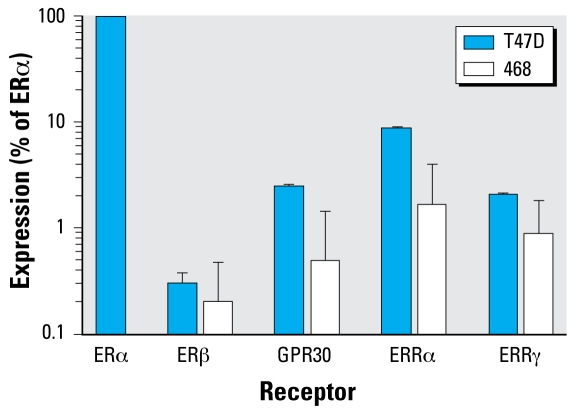
T47D and MDA-MB-468 cells express several types of ERs, as determined by real-time PCR. Both cell lines express ERβ, as well as nonclassical ERs such as GPR30, ERRα, and ERRγ. Data are percentages of ERα expression in T47D cells after corrections for β2M. Values are mean ± SE of five separate experiments.

Using real-time PCR, we compared the expression of several putative ERs in the two cell lines, as percentage of ERα expression in T47D cells. Figure 5 shows that the expression of ERβ was similar in the two cells lines, being < 1% that of ERα. ERRα is the most highly expressed of the alternative receptors in both cell lines, nearing 10% of ERα in T47D cells. The expression levels of GPR30 and ERRγ are similar in T47D cells, with ERRγ slightly higher than GPR30 in MDA-MB-468 cells. ERRβ was undetectable in both cell lines.

### BPA may promote chemoresistance by altering antiapoptotic proteins

**Figure 6:**
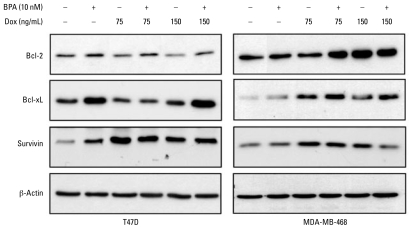
BPA may mediate chemoresistance by altering the expression of prosurvival proteins. Cells were pretreated with 10 nM BPA for 24 hr, followed by exposure to doxorubicin (Dox; 75–150 ng/mL) for an additional 24 hr. Western blots were probed for Bcl-2, Bcl-xL, and survivin, with β-actin serving as a loading control. Shown are representative blots, repeated at least three times.

We next explored the effects of BPA and doxorubicin on the expression of several prosurvival proteins. As shown in Figure 6, treatment of T47D cell with BPA for 24 hr increased both Bcl-2 and Bcl-xL expression. BPA and doxorubicin alone increased expression of survivin, but their combination had no further effect. Both doses of doxorubicin caused a small decrease in Bcl-2 expression, which was partially prevented when cells were pretreated with BPA. In MDA-MB-468 cells, Bcl-2 expression was higher when cells were exposed to 75 ng/mL doxorubicin and BPA compared with 75 ng/mL doxorubicin alone. BPA alone did not increase the expression of Bcl-xL in MDA-MB-468 cells. In both cell lines, Bcl-xL expression was higher in cells treated with 150 mg/mL doxorubicin and BPA compared with 150 ng/mL doxorubicin alone. Survivin expression was increased in both cell types in response to BPA or doxorubicin alone but was not further augmented by their combination.

## Discussion

This is the first report that BPA antagonizes chemotherapeutic agents in both ERα-positive and -negative breast cancer cells. Importantly, unlike some previous studies that have used micromolar concentrations of BPA, we obtained our data using low nanomolar concentrations, which are relevant to human exposure levels. BPA confers chemoresistance to the anticancer drugs doxorubicin, cisplatin, and vinblastine, which act by different mechanisms. As judged by specific ERα/ERβ antagonists, BPA does not appear to mediate its effects through either ERα or ERβ. Given that both cell lines express nonclassical ERs such as GPR30 and members of the ERR family, these could serve as putative BPA receptors. The ability of BPA to alter the expression of Bcl-2 and Bcl-xL suggests a potential mechanism by which it confers chemoresistance in the two breast cancer cell lines.

We postulated that BPA might play a role in chemoresistance based on reports that E_2_ antagonizes anti cancer drugs. For example, taxol-induced cytotoxicity in MCF-7 breast cancer cells was abrogated by 0.1 μM E_2_ (Huang et al. 1997). This was confirmed in a later study implicating JNK activation in the modulation of apoptosis and E_2_ protection (Razandi et al. 2000). In addition, E_2_ antagonizes doxorubicin-induced cytotoxicity in MCF-7 cells (Teixeira et al. 1995). Our data show that BPA protects T47D cells from several anticancer drugs. More unexpected was the effect of BPA on the estrogen-unresponsive MDA-MB-468 cells, raising the prospect that ERα does not mediate the chemoresistant effects of BPA.

The few reports on the effects of BPA on mitogenesis have used the ER-positive MCF-7 cells. Olsen et al. (2003) observed increased MCF-7 cell proliferation in response to BPA, with a relative proliferative potential 60,000 times lower than that of E_2_. Samuelsen et al. (2001) further confirmed such effects of BPA; their MCF-7 data are a prime example of an inverted U-shaped curve that is often observed when treating cells with increasing doses of BPA. In that study, cell proliferation was unchanged in the presence of 10 nM BPA, increased > 40% with 100 nM BPA, peaked with 1 μM BPA, and declined at higher doses. These studies are in agreement with our data, which show an approximate 25% increase in cell viability in T47D cells in response to BPA. Despite the lack of a mitogenic effect of BPA in MDA-MB-468 cells, we observed its ability to antagonize the anticancer drugs with as little as 0.01 nM BPA.

Of particular interest is the ability of BPA to antagonize the cytotoxic effects of three chemotherapeutic agents that induce cell death by different mechanisms. Doxorubicin causes DNA damage by chelating metal ions, generating free radicals, and inhibiting topo-isomerase, thereby blocking transcription (Aubel-Sadron and Londos-Gagliardi 1984). Cisplatin, a platinum-based compound, causes DNA intrastrand cross-linking and inhibits replication (Stewart 2007). Vinblastine acts by interfering with microtubule dynamics, resulting in mitotic arrest and cell death (Toso et al. 1993). As mentioned above, E_2_ protects against microtubule-altering and DNA-damaging drugs (Huang et al. 1997; Teixeira et al. 1995). Thus, drugs with different intracellular targets may have a common mechanism for inducing cell death. Future studies should examine whether BPA protects cells from death ligands that induce apoptosis by binding to proapoptotic death receptors.

BPA weakly competes with 17β-E_2_ in binding to the ER. Using a cell-based transcription assay with a reporter gene, Hiroi et al. (1999) reported that BPA exhibits agonistic activity when signaling through ERβ but has both agonistic and antagonistic activity when interacting with ERα. Whereas T47D cells express both ERα and ERβ, MDA-MB-468 cells have long been used as a model for ER-negative breast cancer. We show that MDA-MB-468 cells express ERβ protein, whose levels can be modulated by treatment with ICI or PHTPP. Like others (Fan et al. 2003; Long and Nephew 2006), we show that ICI rapidly and dramatically degraded the ERα protein, suggesting that the use of ICI is comparable with targeting the receptor with small interfering RNA (siRNA). The finding that BPA exerted its anticytotoxic effects when ERα or ERβ were inhibited suggests that BPA activates a nonclassical ER(s).

Nonclassical ERs include GPR30 and members of the ERR family: ERRα, ERRβ, and ERRγ. BPA binds to GPR30 with a 50% inhibitory concentration (IC_50_) of 630 nM, compared with E_2_ with an IC_50_ of 17.8 nM (Thomas and Dong 2006). Interestingly, ICI binds to GPR30 and acts as an agonist (Prossnitz et al. 2007). Although 17β-E_2_ does not bind to members of the ERR family, ERRs can bind to functional estrogen-responsive elements in ER target genes (Huppunen and Aarnisalo 2004). Among the ERRs, BPA binds strongly to ERRγ, with a dissociation constant (*K*_D_) of 5.5 nM, a much more environmentally relevant dose than that needed to bind to ERα or ERβ (Matsushima et al. 2007). This makes ERRγ the most likely candidate for mediating the protective effects of BPA. Importantly, ERRγ mRNA level was significantly elevated 3.9-fold in breast tumors relative to normal mammary epithelial cells (Ariazi and Jordan 2006). We found that both T47D and MDA-MB-468 cells express GPR30, ERRα, and ERRγ, whereas ERRβ was undetectable. These data identified potential receptors that should be pursued using approaches such as siRNA to determine which receptor(s) mediates the chemoprotective effects of BPA.

The mechanisms underlying chemoresistance include altered expression of proapoptotic/antiapoptotic proteins, increased activity of membrane transporters such as P-glycoprotein, the status of tumor suppressors, and the efficiency of DNA repair processes. The antiapoptotic Bcl-2 and Bcl-xL proteins and survivin, a prosurvival inhibitor of apoptosis, are major players in tumor growth and resistance to cytotoxic insults. Estrogen increases Bcl-2 protein expression in MCF-7 cells, with cells transfected with Bcl-2 antisense twice as sensitive to doxo rubicin treatment in the presence of estrogen compared with controls (Teixeira et al. 1995). Another study suggested that increased Bcl-2 in response to estrogen protects cells from taxol-induced cytotoxicity (Huang et al. 1997). Our data indicate that up-regulation of Bcl-2 and Bcl-xL is a plausible mechanism by which BPA confers resistance to doxorubicin and possibly other anticancer drugs. The survivin data agree with another study that found increased expression of this protein after doxorubicin treatment (Tirro et al. 2006). However, the contributions of survivin are less critical when proteins such as Bcl-2 and Bcl-xL, which are upstream of survivin, mediate survival.

In conclusion, we have shown that low doses of BPA confer chemoresistance to multiple anticancer drugs, possibly by increasing expression of antiapoptotic Bcl-2 proteins. Importantly, we observed the effects of BPA in a cell line lacking ERα, indicating that BPA acts via nonclassical receptors. These data highlight a previously unrecognized function of BPA in cancer management, thereby adding strong support to the growing recognition of the adverse effects of BPA on human health.

## Figures and Tables

**Table 1:** Human gene-specific primers for quantitative real-time reverse-transcriptase PCR.

Gene	Accession no.^a^	Forward primer (5′→3′)	Reverse primer (5′→3′)	Product size (bp)
*ESR1*	NM_000125	CAGGCACATGAGTAACAAAGG	CAAGGAATGCGATGAAGTAGAG	195
*ESR2*	NM_001437	CAGTTATCACATCTGTATGCGG	ACTCCATAGTGATATCCCGA	208
*ESRRA*	NM_004451	ACTGCAGGATGAGCTGG	TGCACAGAGTCTGAATTGG	185
*ESRRB*	NM_004452	CTGGTGTACGCTGAGGA	TACATGGAATCGGAGTTGG	172
*ESRRG*	NM_001438	CATATTCCAGGCTTCTCCA	GACAAGTTCATCCTCAAACGA	122
*GPR30*	NM_001039966	ACGAGACTGTGAAATCCGCAACCA	ATCAGGCTGGAGGTGCACTTGGAA	153
*B2M*	NM_004048	GGCATTCCTGAAGCTGAC	GAATCTTTGGAGTACGCTGG	114

Abbreviations: *ESR1*, ERα; *ESR2*, ERβ; *ESRRA*, ERRα; *ESRRB*, ERRβ; *ESRRG*, ERRγ (all three transcripts). Primer pairs were designed using the PerlPrimer (Marshall 2004) and are all intron-spanning pairs.

a GenBank accession numbers (National Center for Biotechnology Information 2008).
